# Assessment of the Electrolyte Heterogeneity of Tissues in Mandibular Bone-Infiltrating Head and Neck Cancer Using Laser-Induced Breakdown Spectroscopy

**DOI:** 10.3390/ijms25052607

**Published:** 2024-02-23

**Authors:** Philipp Winnand, Klaus Olaf Boernsen, Mark Ooms, Marius Heitzer, Nils Vohl, Matthias Lammert, Frank Hölzle, Ali Modabber

**Affiliations:** 1 Department of Oral and Maxillofacial Surgery, University Hospital RWTH Aachen, Pauwelsstraße 30, D-52074 Aachen, Germany; mooms@ukaachen.de (M.O.); nvohl@ukaachen.de (N.V.); fhoelzle@ukaachen.de (F.H.); amodabber@ukaachen.de (A.M.); 2 Institute of Chemistry and Bioanalytics, University of Applied Sciences Northwestern Switzerland, Hofackerstrasse 30, CH-4132 Muttenz, Switzerland; klausolaf.boernsen@fhnw.ch; 3 Institute of Pathology, University Hospital RWTH Aachen, Pauwelsstraße 30, D-52074 Aachen, Germany; mlammert@ukaachen.de

**Keywords:** head and neck cancer, laser-induced breakdown spectroscopy, potassium, tissue heterogeneity, tissue recognition

## Abstract

Laser-induced breakdown spectroscopy (LIBS) was recently introduced as a rapid bone analysis technique in bone-infiltrating head and neck cancers. Research efforts on laser surgery systems with controlled tissue feedback are currently limited to animal specimens and the use of nontumorous tissues. Accordingly, this study aimed to characterize the electrolyte composition of tissues in human mandibular bone-infiltrating head and neck cancer. Mandible cross-sections from 12 patients with bone-invasive head and neck cancers were natively investigated with LIBS. Representative LIBS spectra (n = 3049) of the inferior alveolar nerve, fibrosis, tumor stroma, and cell-rich tumor areas were acquired and histologically validated. Tissue-specific differences in the LIBS spectra were determined by receiver operating characteristics analysis and visualized by principal component analysis. The electrolyte emission values of calcium (Ca) and potassium (K) significantly (*p* < 0.0001) differed in fibrosis, nerve tissue, tumor stroma, and cell-rich tumor areas. Based on the intracellular detection of Ca and K, LIBS ensures the discrimination between the inferior alveolar nerve and cell-rich tumor tissue with a sensitivity of ≥95.2% and a specificity of ≥87.2%. The heterogeneity of electrolyte emission values within tumorous and nontumorous tissue areas enables LIBS-based tissue recognition in mandibular bone-infiltrating head and neck cancer.

## 1. Introduction

Since their introduction more than 60 years ago, lasers have found their way into a wide range of nonmedical and medical applications [1]. The use of lasers in surgery is widespread and is constantly bringing new innovations to light that aim to facilitate conventional surgery [2]. Technical innovations have already made it possible to perform robot-guided, contact-free osteotomies with lasers instead of using conventional mechanical saws [3,4]. In oncological surgery, intraoperative sampling, in combination with real-time examination of tumor samples using spectrometric inventions, appears to have great potential [5].

Laser-induced breakdown spectroscopy (LIBS) constitutes a direct measurement method [6,7] that has already been used in research for the identification and discrimination of different cancer diseases [8]. Promising results on the LIBS-based differentiation of various hard and soft tissues of the porcine head region have led to theoretical considerations of clinical laser surgery systems with tissue-specific laser ablation [9,10,11,12]. More recently, the potential synergistic effects of laser ablation and laser-assisted tissue detection have been substantiated by combining LIBS systems with tissue-ablating Er:YAG lasers [13,14,15]. However, research efforts on laser surgery systems with controlled tissue feedback in the head and neck region are currently limited to animal specimens and the use of nontumorous tissues [9,10,11,12,13,14,15].

The highly complex anatomy of the head and neck region, defined by a high density of important structures within a limited space, has already led to the increased use of innovative spectroscopic techniques for the real-time assessment of resection margin status in oral cancer [16]. Tumors with bony involvement in particular represent a glaring deficit in the care of head and neck tumor patients because the fact that no methods for rapid bone analysis have been established to date describes a fundamental problem of oncological safety [17,18].

After optimizing the technical requirements of a LIBS-based experimental setup for instrumentation on native sample material and developing a robust method for the evaluation of LIBS spectra [19], we were able to describe the use of LIBS as a promising and innovative possibility for rapid bone analysis for the first time [20]. Subsequently, LIBS-based tracking of the electrolytes calcium (Ca), potassium (K), and sodium (Na), which are involved in the progression of head and neck tumors [21] and in the invasion of tumors into bone [22], was used to record depth profiles at the border of bone-infiltrating head and neck tumors [23]. The further development of LIBS-based rapid bone analysis as a feedback control of a laser surgical system requires algorithms for tissue recognition that can distinguish not only healthy bone but also nerve tissue and fibrosis from tumor stroma and cell-rich tumor areas.

As the next step of the research, the knowledge gained from our previous LIBS studies, which focused on healthy bone and bone-infiltrating oral cancer [20,23], needs to be transferred to other tumorous and nontumorous tissues from mandibular bone-infiltrating head and neck cancer. Accordingly, this study aimed to characterize the electrolyte composition of different tissues with LIBS in preparation for the development of LIBS-based tissue detection algorithms and to enable LIBS for the full-scale evaluation of segmental mandibulectomy specimens. In this study, mandible cross-sections from patients with bone-infiltrating head and neck cancer were natively investigated with LIBS. Representative LIBS spectra of the inferior alveolar nerve, fibrosis, tumor stroma and cell-rich tumor areas were examined for tissue-specific differences and were histologically validated.

## 2. Results

### 2.1. Spectral Differences of Fibrosis, Nerve Tissue, Tumor Stroma, and Cell-Rich Tumor Areas

**Figure 1:**
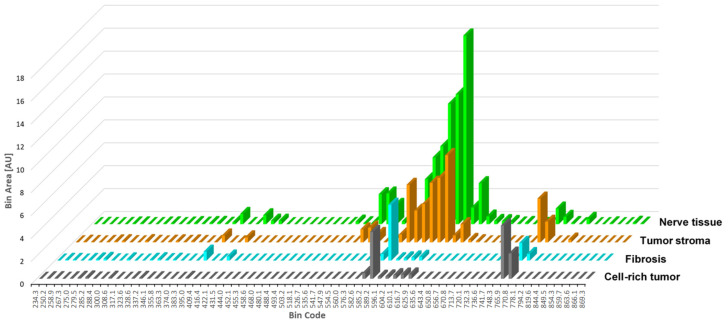
Graphical visualization of tissue-specific differences in the LIBS spectra of nerve tissue (green), tumor stroma (orange), fibrosis (light blue), and cell-rich tumor areas (black). The bar columns show the median values of the peak areas (in arbitrary units [AU]) for the individual wavelengths.

The observation of the median peak areas from all tissue spectra provides the insight that fibrosis, nerve tissue, tumor stroma, and cell-rich tumor tissue can be distinguished on the basis of the Ca-associated wavelengths at 547.89 nm, 554.52 nm, 560.01 nm, 596.05 nm, 604.21 nm, 610.08 nm, 616.66 nm, 625.88 nm, 635.58 nm, 643.38 nm, and 650.8 nm, as well as K-associated wavelengths at 765.86 nm and 770.76 nm. Na-associated wavelengths at 589.14 nm and 819.42 were not helpful in discriminating between different tissues (Figure 1).

**Figure 2:**
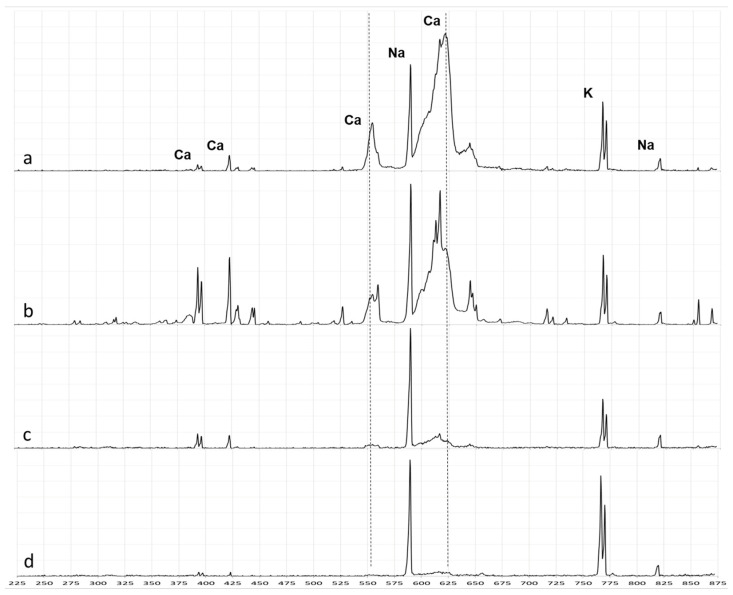
Single representative LIBS spectra of nerve tissue (**a**), tumor stroma (**b**), fibrosis (**c**), and cell-rich tumor areas (**d**). Note that the LIBS spectrum of the tumor stroma (**b**) is twice as intense as the spectra of the nerve tissue (**a**), fibrosis (**c**) and cell-rich tumor areas (**d**).

LIBS spectra from nerve tissue were characterized by high Ca and moderate K intensities (Figure 2a). LIBS spectra from the tumor stroma showed moderate Ca and high K intensities (Figure 2b). LIBS spectra from fibrosis showed high Na and moderate K intensities (Figure 2c). Spectra from cell-rich tumor areas had high Na and K intensities (Figure 2d).

### 2.2. Median Peak Areas of Ca and K in Fibrosis, Nerve Tissue, Tumor Stroma, and Cell-Rich Tumor Areas

The median peak areas of Ca measured 4.973 arbitrary units (AU) in fibrosis, 63.64 AU in nerve tissue, 30.04 AU in tumor stroma, and 1.974 AU in cell-rich tumor, resulting in a significant (*p* < 0.0001) difference between the tissues (Table 1).

**Figure 3:**
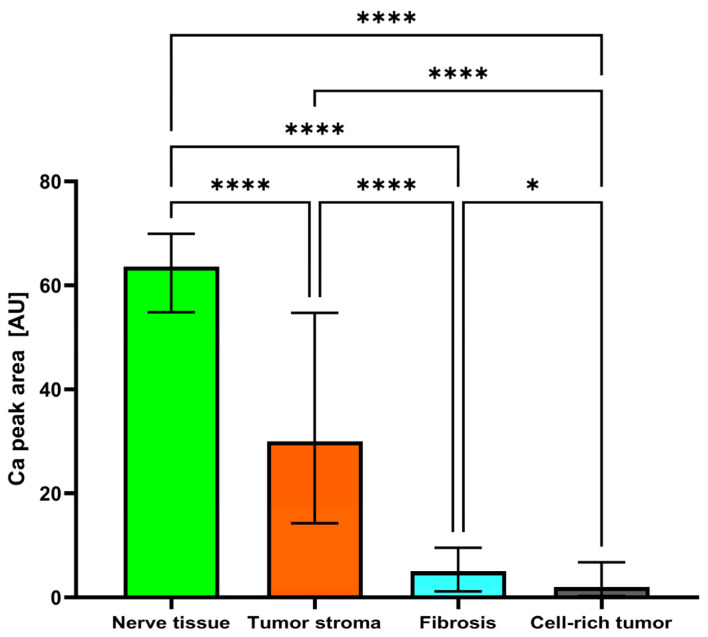
Bar column (median with interquartile ranges [IQR]) of Ca peak areas (in AU) in nerve tissue, tumor stroma, fibrosis, and cell-rich tumor areas. *: *p* < 0.05; ****: *p* < 0.0001.

Ca was significantly (*p* < 0.0001) higher in nerve tissue than in fibrosis, tumor stroma, and cell-rich tumor areas. Ca was significantly (*p* < 0.0001) higher in tumor stroma than in fibrosis and cell-rich tumor areas. Ca was significantly (*p* = 0.0326) higher in fibrosis than in cell-rich tumor areas (Table 2; Figure 3).

**Figure 4:**
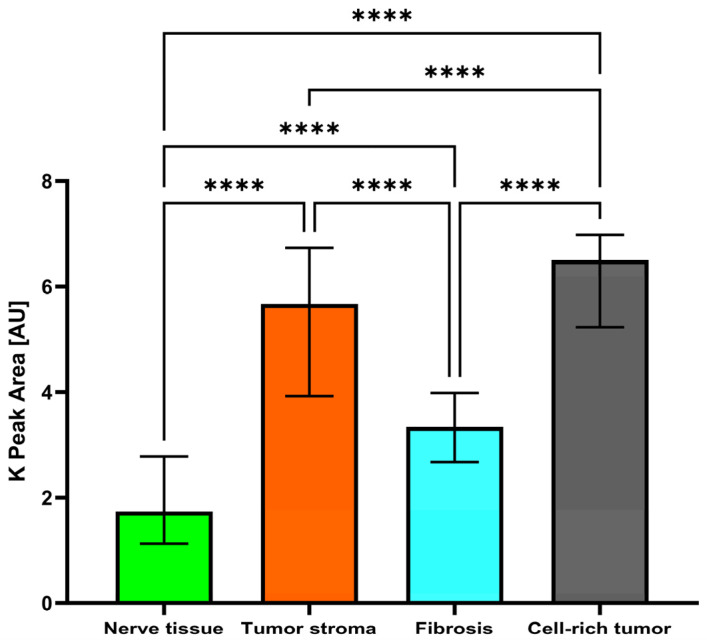
Bar column (median with IQR) of K peak areas (in AU) in nerve tissue, tumor stroma, fibrosis, and cell-rich tumor areas. ****: *p* < 0.0001.

The median peak areas of K measured 3.339 AU in fibrosis, 1.738 AU in nerve tissue, 5.669 AU in tumor stroma, and 6.498 AU in cell-rich tumor areas, resulting in a significant (*p* < 0.0001) difference between the tissues (Table 1). K was significantly (*p* < 0.0001) higher in cell-rich tumor areas than in fibrosis, nerve tissue, and tumor stroma. K was significantly (*p* < 0.0001) higher in tumor stroma than in fibrosis and nerve tissue. K was significantly (*p* < 0.0001) higher in fibrosis than in nerve tissue (Table 2; Figure 4).

### 2.3. Discriminative Power between Different Tissues in Mandibular Bone-Infiltrating Head and Neck Cancer

Using the Ca peak area, fibrosis could be differentiated from nerve tissue with a sensitivity of 93.80% and a specificity of 95.28%, and from tumor stroma with a sensitivity of 79.90% and a specificity of 86.61%. Fibrosis can be distinguished from cell-rich tumor tissue using the K peak areas, with a sensitivity of 84.09% and a specificity of 83.86%. The K peak areas could be used to differentiate between nerve tissue and tumor stroma, with a sensitivity of 81.73% and a specificity of 86.05%. Cell-rich tumor tissue could be distinguished from nerve tissue with a sensitivity of 95.20% and a specificity of 89.53%, and from tumor stroma with a sensitivity of 82.03% and a specificity of 86.36% using the Ca peak areas. Further details are shown in Table 3.

### 2.4. Metric Scale to Differentiate Tissues Based on the LIBS Peak Areas of Ca and K Using ROC Analysis

**Figure 5:**
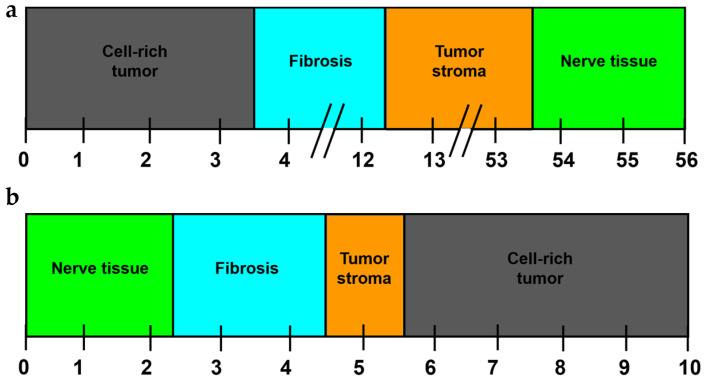
Metric scale to differentiate fibrosis (light blue), nerve tissue (green), tumor stroma (orange), and cell-rich tumor areas (black) based on the LIBS peak areas of Ca (**a**) and K (**b**). The cutoff values were calculated using ROC analysis.

Ca peak areas of 0 to <3.414 indicate cell-rich tumor tissue, while Ca peak areas ≥ 3.414 to <12.21 indicate fibrosis. Ca peak areas ≥ 12.21 to <53.68 are characteristic of tumor stroma. Ca peak areas ≥ 53.68 indicate the presence of nerve tissue (Figure 5a).

K peak areas from 0 to <2.239 indicate nerve tissue, while K peak areas ≥ 2.239 to <4.466 indicate fibrosis. The K peak areas ≥ 4.466 to <5.549 indicate the presence of tumor stroma. K peak areas ≥ 5.549 indicate cell-rich tumor tissue (Figure 5b).

### 2.5. Principal Component Analysis

**Figure 6:**
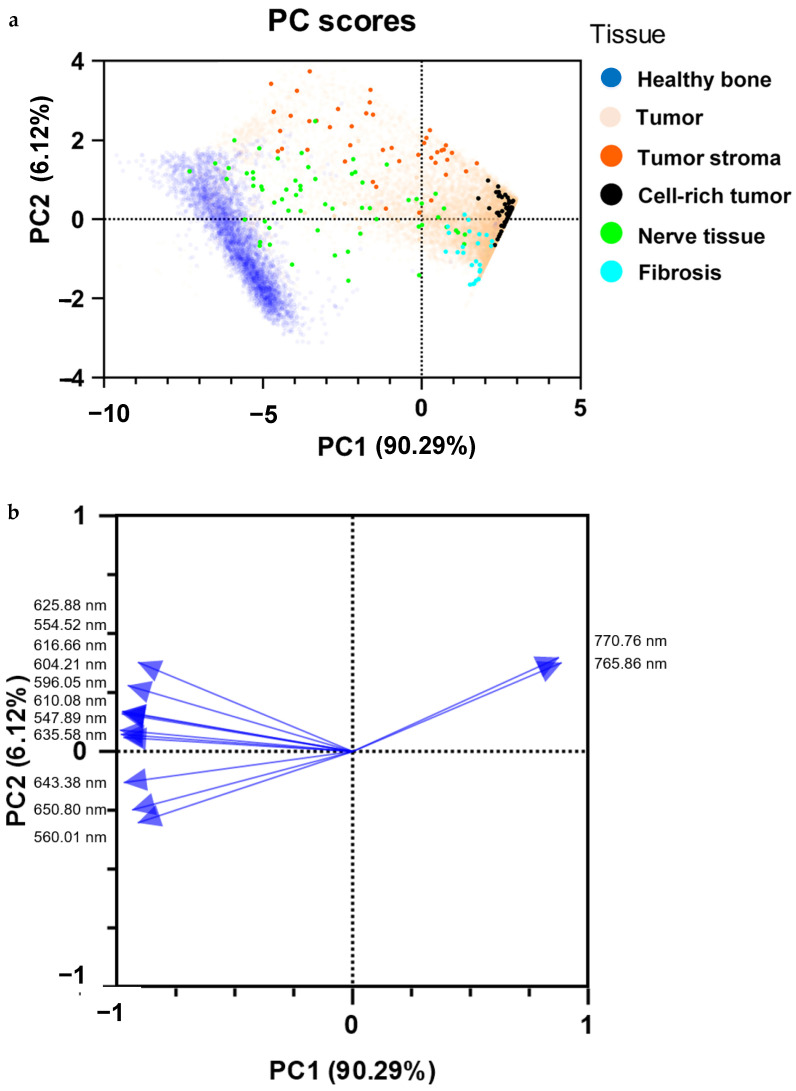
Principal component analysis (**a**) with representative single spectra for visualizing the separability of nerve tissue (green), fibrosis (light blue), tumor stroma (orange), and cell-rich tumor areas (black). Healthy bone (dark blue) and tumor tissue (transparent red) were used for orientation and for fixation of the PCA. The spectra of healthy bone substance and bone-infiltrating tumor tissue were evaluated and published as part of our previous work [20]. Loading plot (**b**) with the most important bins (peak areas of given wavelengths) responsible for the separation of the samples.

The PCA performed with representative individual spectra showed that the spectra of nerve tissue, fibrosis, tumor stroma, and cell-rich tumor areas could be distinguished with sufficient resolution power based on the selected bins (peak areas of given wavelengths) (Figure 6a). The corresponding loading plot demonstrated the dominant role of K (Figure 6b).

**Figure 7:**
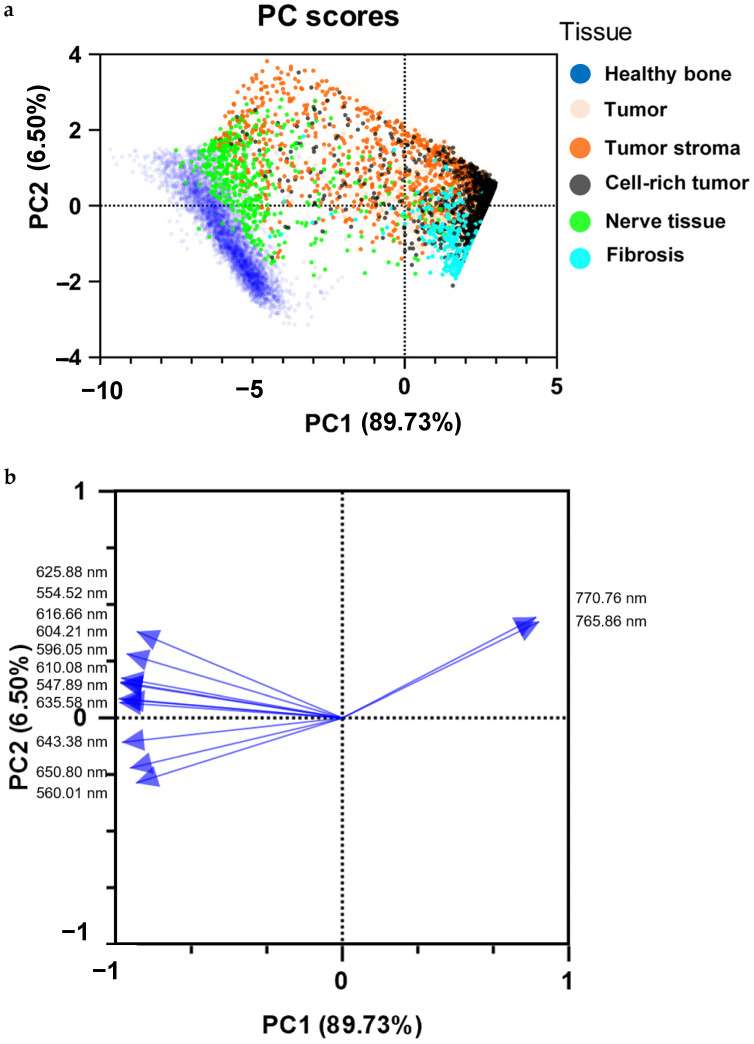
Principal component analysis (**a**) with all spectra for visualizing the separability of nerve tissue (green), fibrosis (light blue), tumor stroma (orange), and cell-rich tumor areas (black). Healthy bone (dark blue) and tumor tissue (transparent red) were used for orientation and for fixation of the PCA. The spectra of healthy bone substance and bone-infiltrating tumor tissue were evaluated and published as part of our previous work [20]. Loading plot (**b**) with the most important bins (peak areas of given wavelengths) responsible for the separation of the samples.

The PCA, which contains all tissue spectra, shows that some of the nerve spectra are very similar to the healthy bone spectra. The spectra of tumor stroma showed a broadly scattered clustering behavior, some of which were close to both the spectra of healthy bone and the tightly clustered tumor cell spectra. The fibrosis spectra were closely clustered and localized far away from the healthy bone spectra (Figure 7a). The corresponding loading plot demonstrated the dominant role of K (Figure 7b).

## 3. Discussion

While conventional mechanical surgery relies on the surgeon’s visual and tactile impressions, laser surgical systems must ensure the remote detection of tissue-specific differences without taking the macroscopic impression of the intraoperative situation into account [15]. Preliminary work available in the literature on LIBS-based ex vivo tissue discrimination between oral hard and soft tissues [9], between fat and nerve [10], and between cartilage and bone [12] demonstrated the potential suitability of LIBS as a spectroscopic-optical sensor for controlled feedback in the context of tissue-specific laser ablation in the head and neck region. The integration of LIBS into a laser system with a tissue-ablating laser also appears technically feasible [13,14,15]. Since the knowledge gained from porcine spectra is not necessarily suitable for the in vivo classification of human tissues [15,24], knowledge of LIBS-based tissue detection on human sample material is required. Against this background, the discrimination of various human, nontumorous and tumorous tissues might be a milestone for the further development of surgical systems with LIBS-based feedback control.

In the present study, the tissue-specific discrimination of the human LIBS spectra was predominantly based on the LIBS emission values of Ca and K. The sufficient LIBS-based tissue separation is exciting, in that tumorous and nontumorous human tissues with comparable densities and consistency were differentiated in this study based on their LIBS signals. Healthy bone substances, whose LIBS spectra were characterized by high Ca and low K signals in our previous work [20,23], were not taken into account in this study for the identification of tissue-specific differences.

The results of our study must be interpreted against the background of biology on the one hand and the details of the study design on the other. In contrast to previous ex vivo LIBS studies, in which the tissue types to be distinguished were isolated from the head of pigs and then individually lasered [9,10,11,12], our LIBS instrumentation was performed on the entire mandible cross-section without extensive sample preparation after lamellar cutting of the segmental mandibulectomy specimens. The fact that the LIBS spectra were thus recorded in realistic examination scenarios required accurate histological annotation of the spectra using a coordinate system. Histological labeling was a major problem in previous publications using spectroscopic optical sensors [25], but it must be considered a decisive strength in this study.

The tissue spectra recorded in this study depict the tissue-specific signal, including the immediate spatial environment within the laser spot diameter of our LIBS-based experimental setup [19]. The inferior alveolar nerve anatomically courses through the mandible within a bony canal [26]. The nerve spectra recorded in this study reveal the location of the nerve in the bony canal by high Ca emission values, which is consistent with our previous publication on Ca-rich LIBS signals in healthy bone substance [20]. Due to the vastly differing handling protocols of the sample material between the current study and other ex vivo LIBS studies [9,10,11,12], comparison of our nerve spectra with the ex vivo spectra of isolated nerves from porcine heads [10] is impossible. The beneficial effect of generating LIBS spectra on the entire native mandible cross-section is also evident in the LIBS spectra of the tumor stroma. In this context, the promotion of bone-invasive tumor growth by tumor stroma and the localization of the tumor stroma within the bone-tumor interface [27,28,29] seem to correspond to increased Ca signals in the spectra of the tumor stroma. However, the Ca-rich LIBS signals in the spectra of the inferior alveolar nerve and tumor stroma make it difficult to discriminate pairwise between these two tissues based on the LIBS detection of Ca.

LIBS detection of K revealed that the tumor stroma and the cell-rich tumor areas had significantly higher K emission values than the nerve tissue and fibrosis. This observation emphasizes the tumor-associated significance of K, which we have already described in the context of discrimination between healthy bone substances and bone-infiltrating tumor tissue [20]. While Wang et al. [30] recently showed that the heterogeneity of elements within tumor cells can be used for LIBS-based discrimination of different tumor cell lines, our study proves that the distribution of elements is also significantly different between different areas within mandibular bone-infiltrating head and neck tumors. Further development of nano-LIBS systems is expected to have great potential for cellular research in the future [31], which could also contribute to addressing current challenges in the diagnosis and treatment of head and neck cancers.

From a surgical point of view, reliable intraoperative differentiation between healthy soft tissue and bone-infiltrating tumor tissue represents the next milestone regarding the oncological safety of a LIBS-based tissue recognition system. The LIBS-based differentiation between fibrosis and tumor stroma is performed with higher accuracy by detecting Ca rather than K, while the discrimination between fibrosis and cell-rich tumor tissue is better with K than with Ca. Differentiation between fibrosis and tumorous areas must be a central part of LIBS-based tissue recognition, as fibrosis is the most common host cell reaction in mandibular invasion by head and neck tumors [32]. Ultimately, LIBS-based discrimination between Na- and K-rich fibrosis and tumor tissue in the head and neck region remains a challenge. LIBS studies on liver fibrosis have already indicated that other wavelengths and other electrolytes might also be involved in the progression of fibrotic conditions [33], which should be considered for future LIBS studies and, in particular, for improving tissue discrimination of fibrosis versus tumorous tissues. The LIBS-based cell type differentiation shown in this work is a first approach using only selected electrolytes that contributed most to the separation between tissues. These included Ca with calcium oxide (CaO) and calcium hydroxide (CaOH) emission lines and K, while Na was less suitable for capturing tissue heterogeneity in this study. Further discussion regarding the wavelengths used in this study is already part of previous publications on calibration experiments with K on native sample material [19] and previous works on the use of LIBS as a rapid bone analysis technique [20] and LIBS-based depth profiling [23].

The results of the current study may contribute to a better understanding of the complexity in the dysregulation of Ca, K and Na ion channels in head and neck cancer cells [21]. While K and Na are involved in the invasiveness, progression and proliferation of head and neck cancer cells [21,34], Ca channels are involved in the differentiation of osteoblasts and osteoclasts [22] and thus, orchestrate the invasion of head and neck tumors into bone [22,35], as recently discussed [23]. Since the present study provides an indicative overview of electrolyte levels within tumorous and nontumorous tissues in mandibular bone-infiltrating head and neck cancer, future studies are needed to investigate the relationship of K, Na, and Ca levels in tumor tissues with more advanced statistical methods in more detail. A LIBS-based rapid tissue analysis technique for bone-infiltrating head and neck cancer must finally be able to detect all potential pathways of tumor spread into and through the bone. The clinical relevance arises from the high rate of unexpected bone invasion in oral squamous cell carcinomas in proximity to the mandible [36]. Direct tumor invasion through the bone is the most common scenario [32] and can be accurately covered by LIBS-based differentiation of healthy bone and bone-infiltrating tumor tissue, as previously shown [20]. However, 20% of bone-infiltrating tumors grow along the inferior alveolar nerve [32], and 43% of all bone-infiltrating tumors exhibit radiological involvement of the bony nerve canal, which is associated with poor survival [37]. According to the interpretation of the current study results, LIBS-based tissue discrimination appears to ensure the differentiation between cell-rich tumor areas and the inferior alveolar nerve with a sensitivity of ≥95.2% and a specificity of ≥87.2%, based on the intracellular detection of Ca and K. In the concrete intraoperative implementation of LIBS-guided feedback control, the performance of pairwise tissue discrimination would ultimately have to be summarized on a metric scale, as shown in this study. In this way, the measured electrolyte emission values could be used to draw conclusions about the respective lasered tissue in real time.

As with our previous LIBS studies [19,20,23], minor limitations must also be considered in this study. The number of patients limited the number of spectra available in this study. Representative spectra were selected and analyzed without advanced and automated statistical methods, which may have caused selection bias. Due to ethical reasons, the sample material was lasered ex-vivo. For future research, tumor-induced animal models are recommended to obtain in-vivo spectra. Using more advanced and automated statistical methods on larger LIBS datasets are suggested for future research to analyze tissue-specific differences even better.

The robust evaluation of the LIBS spectra based on biological principles, the histological annotation of the LIBS spectra using a coordinate system and the realistic LIBS instrumentation on native tumor-infiltrated, human mandibular bone sections are the strengths of this study. The differentiation of tumorous and nontumorous tissues trained in this way with LIBS is a novelty and represents a significant addition to our previous work [19,20,23] and to the available literature [9,10,11,12].

Regarding possible future application potential, this work demonstrates the strengths and weaknesses of LIBS-based tissue recognition in human tissues of tumorous and nontumorous origin, which represents a significant contribution to our previous work [20,23] and the available literature [9,10,11,12]. When considering our results, the high technical sensitivity of LIBS in the instrumentation of native sample materials must be considered [19]. As discussed in a previous publication [20], the water content of the samples and the associated quenching effects can have an influence on the measured LIBS signal [38,39], especially when LIBS instrumentation is performed on native sample material. In this study, the quenching effect was anticipated by absorbing the intercellular fluid of the native sample material with round filter types prior to the LIBS experiments. In addition, the technical requirements of the LIBS setup and the evaluation method were previously optimized for reproducible LIBS measurements with high single-shot sensitivity on native sample material [19]. The knowledge gained in this study will be used for further training and optimization of LIBS-based tissue detection algorithms, for which machine learning algorithms could be beneficial, as shown by others [40,41,42].

As a potential application of the study findings, LIBS could be used in the future to detect the various components within the tumor microenvironment that are known to contribute to radioresistance [43]. In this way, LIBS could potentially support radiation oncologists in the development of complex treatment plans and improve the effectiveness of radiotherapy. Regarding the development of clinical multimodal laser surgery systems, optical coherence tomography (OCT) provides promising results for additional real-time feedback and depth control of laser shots [13,44].

## 4. Materials and Methods

### 4.1. Ethical Approval

The Ethics Committee of the Medical Faculty of RWTH Aachen University, Germany, granted ethical approval to conduct the study (EK 126/21). Written informed consent was obtained from 12 patients with bone-infiltrating oral cancers prior to performing a segmental mandibulectomy. The collection of the resection specimens was carried out together with the RWTH centralized Biomaterial Bank (RWTH cBMB) of the Medical Faculty of the RWTH Aachen University. The current version of the Declaration of Helsinki was followed in the conduct of this study.

### 4.2. Preparation of Segmental Mandibulectomy Specimens

The segmental mandibulectomy specimens were prepared as described previously [20,23]. After macroscopic examination, the segmental mandibulectomy specimens were natively sawn into cross-sections with a thickness of 4 mm. Bony cross-sections that showed macroscopically tumor infiltration into the bone were selected for LIBS experiments. Routine histology was not affected. The bony cross-sections were stored at −20 °C and thawed immediately before the LIBS experiments. Then, the bony cross-sections were carefully cleaned with physiological NaCl solution (Fresenius Kabi, Bad Homburg, Germany). The mandibular cross-sections were placed on ash-free filter paper (Binzer and Munktell Filter, Battenberg, Germany) until LIBS instrumentation. In this way, the intercellular fluid was absorbed from the samples and the water content of the samples was reduced.

### 4.3. Experimental Setup

The Advanced Osteotomy Tools (AOT) LIBS system (Advanced Osteotomy Tools (AOT) AG, Basel, Switzerland) was used for the LIBS experiments. As previously described [20,23], the native cross-sections were instrumented with 30 pulses from a Q-switched Nd:YAG laser (λ = 1064 nm) at each laser position. Further technical details were presented in a previous publication [19]. The LIBS instrumentation took 30 min for each bony cross-section.

**Figure 8:**
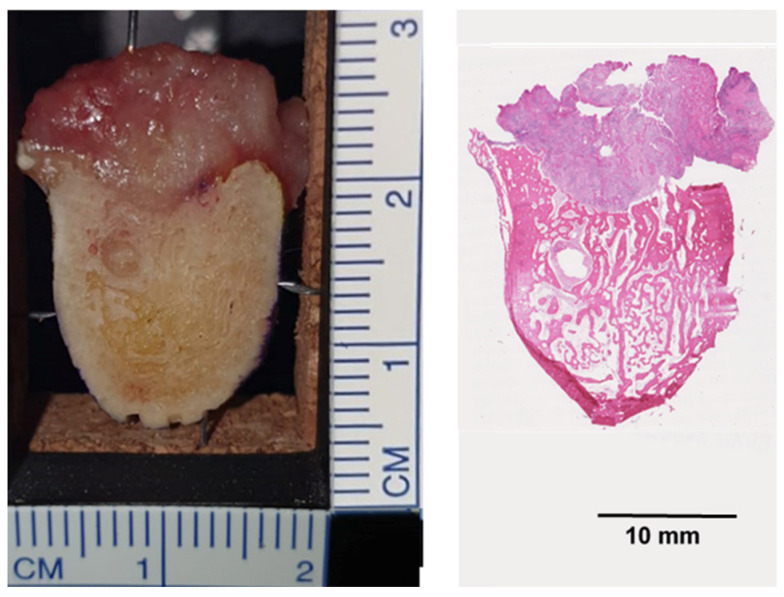
Native mandibular cross-section with tumor infiltration into the bone (**left**) and the corresponding H&E section after performing routine histology (**right**).

To histologically validate the lasered positions on the bony cross-sections, markings were made in the outer cortical layer of the mandibular cross-sections using a thin separating disc. After LIBS instrumentation, routine histology was performed. This meant that the lasered cross-sections were decalcified in ethylenediamine tetraacetic acid (EDTA) (A. Menarini Diagnostics, Berlin, Germany), embedded in paraffin, cut, and hematoxylin and eosin (H&E) (Morphisto, Offenbach, Germany) stained (Figure 8). Using bone markings, the lasered positions were assigned to the corresponding positions on the H&E sections. The LIBS spectra of fibrosis, nerve tissue, tumor stroma, and cell-rich tumor areas were finally validated by a pathologist. Nerve spectra were generated from the inferior alveolar nerve. Only spectra of peripheral neural tissue in which the inferior alveolar nerve was not histologically infiltrated by the tumor were used. Fibrosis was defined as nontumor stroma which contained collagen-rich matrix, fibroblasts and inflammatory cells outside the tumor boundaries, without any neoplastic cells. LIBS spectra of tumor stroma were acquired at spots containing collagen-rich matrix, fibroblasts and inflammatory cells within the tumor boundaries, possibly admixed with minor amounts of neoplastic cells. LIBS spectra of cell-rich tumor were acquired at spots with predominance of neoplastic cells.

### 4.4. Spectra Processing

Recording of the spectra was performed without averaging. As in our previous work [19,20,23], this was the best way to mimic the natural inhomogeneity of the biological tissue. While averaging the spectral data might have been useful to increase the signal-to-noise ratio, it would also have led to a leveling of cell differences. Therefore, averaging the spectra data would not have met the objectives of this study. On top of that, averaging of the LIBS spectra was not needed because of the high single-shot sensitivity of the experimental LIBS setup, which has been demonstrated previously [19]. The spectra were processed using the AOT proprietary data processing method. This included subtraction of the spectrometer baseline, normalization to the base peak intensity, and peak area calculation, as previously described [19,23]. The spectra were processed with a Microsoft Excel (Microsoft, Redmond, Washington, USA) macro.

Representative spectra of fibrosis, nerve tissue, tumor stroma, and cell-rich tumor areas were identified using the highest tissue-specific median values of all 71 bins. Spectral differences between fibrosis, nerve tissue, tumor stroma, and cell-rich tumor areas were observed at 547.89 nm, 554.52 nm, 560.01 nm, 596.05 nm, 604.21 nm, and 610.08 nm, as well as 616.66 nm, 625.88 nm, 635.58 nm, 643.38 nm, and 650.8 nm, which are associated with Ca, and at 765.86 nm and 770.76 nm, which are associated with K. Na-associated wavelengths were localized at 589.14 nm and 819.42 nm. The Na-associated wavelengths were not useful for distinguishing between different tissues, which could be due to the cleaning of the samples with NaCl solution prior to the LIBS measurements.

Principal component analysis (PCA) was first performed with representative single spectra and then with all spectra using these 13 bins (peak areas of given wavelengths). The separation of fibrosis, nerve tissue, tumor stroma, and cell-rich tumor areas was visualized as scatter plots of the first two principal components (PC). For orientation, the PCA was fixed using the spectra of healthy bone substances and bone-infiltrating tumor tissue, which were evaluated and published as part of our previous work [20].

### 4.5. Statistical Analysis

Peak areas of Ca and K were given as median values with interquartile ranges. The Kruskal–Wallis test was used to test for differences in Ca and K peak areas between fibrosis, nerve tissue, tumor stroma, and cell-rich tumor areas. Dunn’s post hoc test was used for pairwise comparison, and the *p*-values were adjusted for multiple testing.

Receiver operating characteristics (ROC) analysis was used to test the discriminatory power of the Ca and K peak areas between two tissue types. The area under the curve (AUC) was calculated for diagnostic accuracy. Calculating the Youden index [45], the optimal threshold value was theoretically determined.

*p*-values < 0.05 were considered statistically significant. Visualization of the bar columns and the principal component analysis, as well as the statistical analysis, were performed using GraphPad Prism 9 (GraphPad Software, San Diego, CA, USA).

## 5. Conclusions

The heterogeneity of electrolyte emission values within tumorous and nontumorous tissue areas enables LIBS-based tissue recognition in mandibular bone-infiltrating head and neck cancer. As a rapid and direct tissue detection technique, LIBS detects both direct tumor invasion into the bone and is able to differentiate between the inferior alveolar nerve and cell-rich tumor areas in bone-infiltrating head and neck tumors. In the future, LIBS-guided feedback control could make an enormous contribution to tissue-specific laser ablation and the future establishment of clinical laser surgery systems. Future research efforts need to confirm our observations in vivo.

## Figures and Tables

**Table 1:** Comparison of median peak areas of Ca and K between fibrosis, nerve tissue, tumor stroma, and cell-rich tumor areas.

Variable	Fibrosis (n = 254 Spectra)	Nerve Tissue (n = 516 Spectra)	Tumor Stroma (n = 821 Spectra)	Cell-Rich Tumor (n = 1458 Spectra)	*p* Value
Ca	4.973 (8.369)	63.64 (15.07)	30.04 (40.46)	1.974 (6.47)	<0.0001
K	3.339 (1.31)	1.738 (1.652)	5.669 (2.812)	6.498 (1.75)	<0.0001

Parameters are indicated as median values. The Ca peak areas were summed at 547.89 nm, 554.52 nm, 560.01 nm, 596.05 nm, 604.21 nm, 610.08 nm, 616.66 nm, 625.88 nm, 635.58 nm, 643.38 nm, and 650.8 nm. The K peak areas were summed at 765.86 nm and 770.76 nm. Differences between parameters were analyzed using the Kruskal–Wallis test. Significant *p* values are shown in bold.

**Table 2:** Pairwise comparison of the median peak areas of Ca and K.

Variable	Comparison	*p* Value (Ca)	*p* Value (K)
Fibrosis vs.	Nerve tissue	**<0.0001**	**<0.0001**
	Tumor stroma	**<0.0001**	**<0.0001**
	Cell-rich tumor	**0.0326**	**<0.0001**
Nerve tissue vs.	Fibrosis	**<0.0001**	**<0.0001**
	Tumor stroma	**<0.0001**	**<0.0001**
	Cell-rich tumor	**<0.0001**	**<0.0001**
Tumor stroma vs.	Fibrosis	**<0.0001**	**<0.0001**
	Nerve tissue	**<0.0001**	**<0.0001**
	Cell-rich tumor	**<0.0001**	**<0.0001**
Cell-rich tumor vs.	Fibrosis	**0.0326**	**<0.0001**
	Nerve tissue	**<0.0001**	**<0.0001**
	Tumor stroma	**<0.0001**	**<0.0001**

Differences between parameters were analyzed using Dunn’s post hoc test for pairwise testing. Significant *p* values are shown in bold.

**Table 3:** ROC analysis for discrimination between two tissue types.

Variable	AUC (95% CI)	*p* Value	Cutoff	Sensitivity	Specificity
Fibrosis vs. Nerve tissue					
Ca	0.9851 (0.9789–0.9913)	**<0.0001**	>19.92	0.9380	0.9528
K	0.8157 (0.7866–0.8448)	**<0.0001**	<2.239	0.6628	0.8701
Fibrosis vs. Tumor stroma					
Ca	0.8923 (0.8716–0.9129)	**<0.0001**	>12.21	0.7990	0.8661
K	0.7972 (0.7700–0.8245)	**<0.0001**	>4.466	0.6760	0.8346
Fibrosis vs. Cell-rich tumor					
Ca	0.5963 (0.5594–0.6332)	**<0.0001**	<3.414	0.6125	0.6181
K	0.9161 (0.8994–0.9328)	**<0.0001**	>4.499	0.8409	0.8386
Nerve tissue vs. Tumor stroma					
Ca	0.7978 (0.7735–0.8220)	**<0.0001**	<53.68	0.7430	0.7713
K	0.9304 (0.9179–0.9429)	**<0.0001**	>3.428	0.8173	0.8605
Nerve tissue vs. Cell-rich tumor					
Ca	0.9795 (0.9744–0.9846)	**<0.0001**	<34.02	0.9520	0.8953
K	0.9776 (0.9723–0.9830)	**<0.0001**	>3.500	0.9554	0.8721
Tumor stroma vs. Cell-rich tumor					
Ca	0.8977 (0.8847–0.9107)	**<0.0001**	<9.495	0.8203	0.8636
K	0.6301 (0.6057–0.6545)	**<0.0001**	>5.549	0.7140	0.4872

The data are presented as area under the curve (AUC) with 95% CI and *p*-value, optimal threshold value, and sensitivity and specificity corresponding to ROC analysis and calculation of Youden Index (YI) separately for prediction of fibrosis, nerve tissue, tumor stroma, or cell-rich tumor areas (fibrosis vs. nerve tissue, fibrosis vs. tumor stroma, fibrosis vs. cell-rich tumor areas, nerve tissue vs. tumor stroma, nerve tissue vs. cell-rich tumor areas, and tumor stroma vs. cell-rich tumor areas) based on K and Ca. The discriminatory power of Ca was calculated using the peak areas at 547.89 nm, 554.52 nm, 560.01 nm, 596.05 nm, 604.21 nm, 610.08 nm, 616.66 nm, 625.88 nm, 635.58 nm, 643.38 nm, and 650.8 nm. The discriminatory power of K was calculated using the peak areas at 765.86 nm and 770.76 nm. Significant *p* values are shown in bold.

## Data Availability

The data presented in this study are available on request from the corresponding author.
